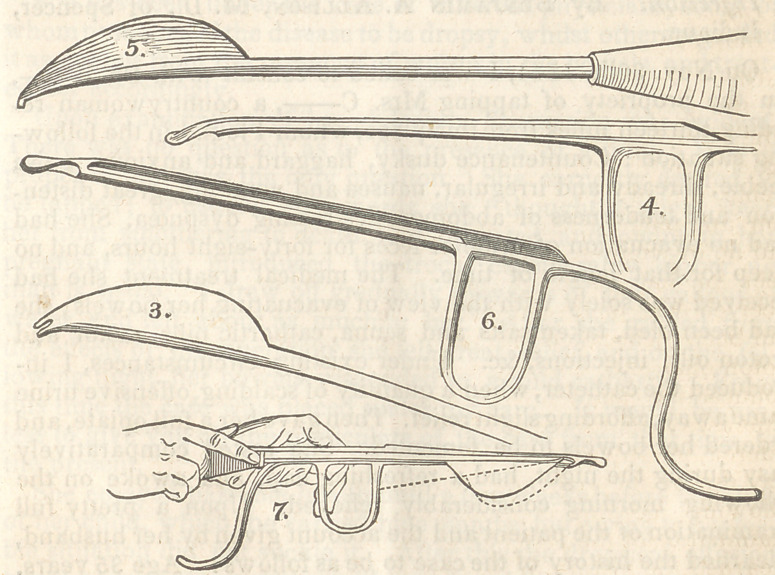# On the Director and Cutting Gorget

**Published:** 1846-06

**Authors:** W. R. Wilde

**Affiliations:** Dublin, (Ireland,) Editor of the Dublin Quarterly Journal, etc.


### MEDICAL EXAMINER,

AND

### RECORD OF MEDICAL SCIENCE.

NEW SERIES.—No. XVIII.—JUNE, 1846.

### ORIGINAL COMMUNICATIONS.

*On the Director and cutting Gorget.* By W. R. Wilde, M.D., of Dublin, (Ireland,) Editor of the Dublin Quarterly Journal, etc.

To the Editor of the Medical Examiner.

Sir,—In the July and November numbers of the Medical Examiner of last year, I perceive that a discussion is pending between two of your countrymen, Professor Mettauer and Mr. Elliott, with regard to the priority of inventing a peculiar form of Director and cutting Gorget for performing the operation of Lithotomy. Conceiving that an injustice has been done (I am willing to hope unintentionally) to three distinguished Irish surgeons, two long since gathered to their fathers, and one the present father of the Surgical Profession in Dublin, I beg to solicit the insertion of the following observations in your valuable journal—preferring the pages of a native periodical, as being more likely to effect the object I have in view, than either those of my own, or any of those published in Great Britain.

The question at issue between Messrs. Mettauer and Elliott, is as to the invention of two instruments; a straight director to be introduced into the bladder (after the external incision has been effected with the knife) by means of a beak, inserted into the groove of the ordinary staff;—and a cutting gorget-like knife with such a mechanical contrivance at its extremity as shall retain its point moving in a line with a director, while with its curved edge the prostate and bladder are divided.

I shall not enter into a description of the rival instruments of your countrymen, because the subject is no doubt fresh in the recollection of your readers, and because, in principle and design, they are essentially the same ; nor will I in this notice entertain the question of the value of these instruments, or the so-called superiority of the operation in which they are used, over other and more generally employed modes of operating in Europe, and Great Britain in particular.

In the year 1750, Mr. Daunt, an eminent surgeon in this city, contrived a conductor and lithotome, figs. 1 and 2, which were considered at that time the best instruments that had been invented for obviating the disadvantages of the cutting gorget of Hawkins, then much employed in this country, and of securing "to the operator a certainty of dividing the membraneous part of the urethra, prostate gland and neck of the bladder, without putting his dexterity to any severe trial." * * * "Having then opened the membraneous part of the urethra, the operator introduces the conductor along the groove of the staff into the bladder; he then withdraws the staff and takes the conductor in his left hand. Having introduced his two fore-fingers into the handle A, he places his thumb over the bow of the instrument B, which gives him an entire firmness as to the rest of the operation. He then lateralises the conductor by the pronation of his wrist, and takes the lithotome and engages it on the crest of the conductor, and finishes the operation by running the lithotome along the crest. Having arrived at the extremity of the conductor, he withdraws the knife along the crest, and then introduces the forceps on the conductor, which withdrawn, he proceeds to the extraction of the stone."*

In 1754, these instruments with a description of them were sent to the celebrated Morand, who presented them to the Royal Academy of Surgery in Paris, who appointed a committee to decide on their merits, and the "Academie des Sciences" published an account of them in their transactions.

On the 22nd February, 1755, M. Audouillé, the Secretary of Foreign Correspondence of the French Academy, addressed to Mr. Daunt a letter expressing the favourable opinion which that body entertained of his new invention, but likewise stating that the committee suggested that if the point of the instrument (lithotome) was made a little broader, and more convex, and the base narrower, it would, they conceived, effect the desired object with greater facility.

Several years afterwards, but during (I believe) the life time

* Observations on the Hydrocele, &c., to which is added a comprehensive view of the different methods of cutting for the stone, &c. By William Dease, Dublin, 1702. 8vo. pp. 149.

of Daunt, the elder Dease, in whose hands the correspondence of the French Academy was placed by Mr. Daunt, acting upon the suggestions of the Parisian Surgeons, modified the instrument, and gave it the form represented in fig. 3.

Mr. Dease likewise gave the staff which he used a greater curve than the generality of instruments have.

Fig. 1 represents the original conductor, and Fig. 2 the lithotome of Mr. Daunt—

With these instruments, Mr. Morris, a Surgeon of Mercer's Hospital in this city, extracted in the year 1773 from Mr. Orford, then aged 56, one of the largest calculi that, as far as I am aware, have been recorded as having been extracted entire by the lateral operation. It weighed 15 ounces and a half, and is figured in Mr. Dease's book.

Figs 3 and 4 are the instruments of Mr. Dease.

In 1807, the present Mr. Peil of this city, and in his day one of the most eminent Lithotomists in Great Britain, improved upon the inventions of his predecessors by substituting a groove in the director instead of the keel which it formerly possessed, and adapting to this one beak to the end of the lithotome instead of the two possessed by the instruments of Messrs. Daunt and Dease.

Mr. Peil published an account of these instruments in the Dublin Medical and Physical Essays for March, 1807, and also figured the instruments which I have represented in figs. 5 and 6. To this paper I would refer your readers, as well as to my Preface to the new series of the Dublin Quarterly Journal of Medical Science for February, 1846, p. 33.

Fig. 7, represents the mode of application of the instruments, and the dotted line shows the manner in which the Lithotome may be depressed so as to enlarge the opening of the Bladder.

I have the honor to be, Sir,

Your obedient serv't.

W. R. Wilde.

Dublin, 16th April, 1846.

Note—The Figures are reduced to about three-sevenths of the size in the drawings sent by the author, to adapt them to our page.—Ed.

## Figures and Tables

**Fig 1. f1:**
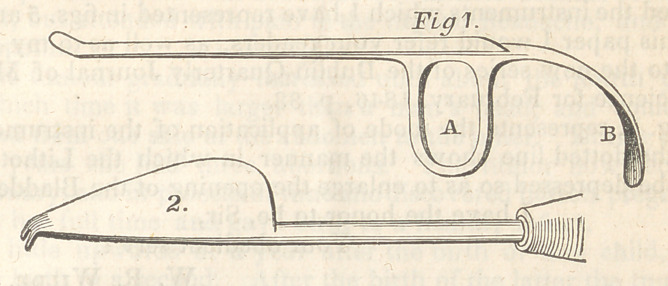


**Figure f2:**